# Association between vitamin D insufficiency and depressive symptoms, and functional disability in community-dwelling Brazilian older adults: results from ELSI-Brazil study

**DOI:** 10.1038/s41598-024-62418-z

**Published:** 2024-06-17

**Authors:** Ana Maria Martins dos Santos, Vanessa Pereira Corrêa, Núbia Carelli Pereira de Avelar, Cesar de Oliveira, Ione Jayce Ceola Schneider

**Affiliations:** 1 Graduate Program in Rehabilitation Sciences, Federal University of Santa Catarina, Rodovia Governador Jorge Lacerda, 3205, Jardim das Avenidas, Araranguá, SC 88906-072 Brazil; 2 Graduate Program in Public Health, Federal University of Santa Catarina, Rodovia Governador Jorge Lacerda, 3205, Jardim das Avenidas, Araranguá, SC 88906-072 Brazil; 3 Department of Health Science, Federal University of Santa Catarina, Rodovia Governador Jorge Lacerda, 3205, Jardim das Avenidas, Araranguá, SC 88906-072 Brazil; 4 Department of Epidemiology and Public Health, University College London, 1-19 Torrington Place, London, WC1E 6BT UK

**Keywords:** Activities of daily living, Aging, Mental health, Public policy, Biomarkers, Geriatrics, Quality of life

## Abstract

Low serum 25(OH)D levels (< 30 nmol/L) have been associated with increased depressive symptom scores over time, and it is believed that functionality may play a mediating role in the relationship between 25(OH)D and depressive symptoms. To comprehend the association between these factors could have significant implications for public health policy. The aim of this study was to verify the association between simultaneous vitamin D insufficiency and depressive symptoms, and functional disability in community-dwelling older adults. This was a cross-sectional study with data from the Brazilian Longitudinal Study of Aging (ELSI-Brazil), collected between 2015 and 2016. The outcomes were functional disability assessed through basic activities of daily living (ADL) and instrumental activities of daily living (IADL). The exposures were vitamin D insufficiency (< 30 nmol/L) and depressive symptoms (≥ 4 points in 8-item version of the Center for Epidemiological Studies-Depression). Crude and adjusted Poisson regression was performed to estimate associations. A total of 1781 community-dwelling older adults included in this study, 14.6% had disability in ADL and 47.9% in IADL; 59.7% had vitamin D insufficient levels, and 33.2% depressive symptoms. The concomitant presence of vitamin D insufficient and depressive symptoms increased the prevalence of ADL by 2.20 (95% CI: 1.25; 3.86) and IADL by 1.54 (95% CI: 1.24; 1.91), respectively. Therefore, preventive strategies to keep older adults physically and socially active, with a good level of vitamin D, are essential to avoid depression and functional disability.

## Introduction

Aging is a normal development process, and it occurs in an irreversible, progressive, and natural manner^1^. An important marker of healthy aging is functional capacity, which refers to a person’s ability to perform essential daily activities for self-care and independent living. Assessing an older adult’s function involves evaluating their ability to perform basic activities of daily living (ADL) and more complex activities, known as instrumental activities of daily living (IADL). When they cannot perform these tasks independently, it indicates a loss of function, requiring daily assistance. This often increases their care needs, impacting their quality of life and increasing the demand for healthcare services^1–3^.

Functional disability can be associated with genetic inheritance and individual and behavioural characteristics, and it is related to autonomy in later life^4–7^. In addition to these risk factors, depressive symptoms^8–10^ and vitamin D insufficiency^11–16^ are also associated with functional disability. Depression is the second most common cause of functional loss and psychosocial disability in the general population. This is characterized by the presence of depressive symptoms. According to the World Health Organization (WHO), at least 10.0% of older adults suffer from this condition, which is one of the disorders that most compromise the quality of life of this population^17–19^.

Depressive symptoms and impairment in functionality reinforce each other, progressing to disability. Additionally, other illnesses may emerge as disability and depression set in^20^. Some physical illnesses resulting from depressive symptoms are explained by the decline in the immune system, as the neurological, endocrine, and immune systems are related to the regulatory functions of the organism in response to internal and external stimulation^21–23^.

It is also known that vitamin D insufficiency is prevalent in the older adults, affecting more than 50% of globally population^24^ and this may imply psychiatric and neurological disorders^25^, as individuals show greater cognitive impairment, depressive symptoms, and anxiety^26^. There is a belief that vitamin D receptors, promoted in brain regions where serotonin genes are present, play a role in these manifestations^27,28^. In addition, the decrease in serum levels of vitamin D in individuals with depressive symptoms can be seen in a secondary way, because due to the symptoms, they spend more time at home, exposing themselves less to the sun and with a poor diet^27^. Vitamin D insufficiency is associated with decreased physical performance, reduced mobility, deterioration of muscle function, and increased incidence of falls and fractures in the older population^11–13, 26^.

Functional capacity is associated with vitamin D insufficiency, as well as the presence of depressive symptoms, but the interrelationship between depressive symptoms and vitamin D and how it affects functional capacity is poorly explored. It is known that low serum 25(OH)D levels (< 30 nmol/L) are associated with increased depressive symptom scores, and it is suggested that functionality may have a mediating role in the relationship between 25(OH)D and symptoms depressive. However, vitamin D supplementation does not show an association with the reduction of depressive symptoms and improvement in physical functioning^29^.

The investigation and comprehension of the association between vitamin D insufficiency, depressive symptoms, and functional disability among community-dwelling older adults could have significant implications for public health policies. Although there is still a gap in the literature regarding their association, these are preventable conditions. The aim of the present study was to verify the association between simultaneous vitamin D insufficiency and depressive symptoms, and functional disability in community-dwelling older adults. Our hypothesis was that the combination of vitamin D insufficiency and depressive symptoms increases the prevalence of functional disabilities in the older adults.

## Methods

### Study design

This was a cross-sectional study with data from the Brazilian Longitudinal Study of Aging (ELSI-Brazil). A population-based longitudinal study, representative of the non-institutionalized Brazilian population aged 50 and over. The baseline survey was carried out between 2015 and 2016, in 70 municipalities in the 5 Brazilian geographic regions. To ensure that the sample represents the urban and rural areas of municipalities of all sizes, the sample was recruited using a design with selection stages, combining stratification of the primary sampling units (municipalities), census sectors, and households. Detailed information on design, recruitment methods, and topics covered are available in the study by Lima-Costa et al.^30^.

The ELSI-Brazil project was approved by the Research Ethics Committee of the Oswaldo Cruz Foundation (FIOCRUZ), Minas Gerais (CAAE: 34649814.3.0000.5091). All participants or legal guardian signed separate informed consent forms before they participated in the study. This study was carried out according to the Resolution n° 466/2012, of the National Council of Health, from the Brazilian Ministry of Health, and principles of the Declaration of Helsinki (1964).

### Population and sample

**Figure 1:**
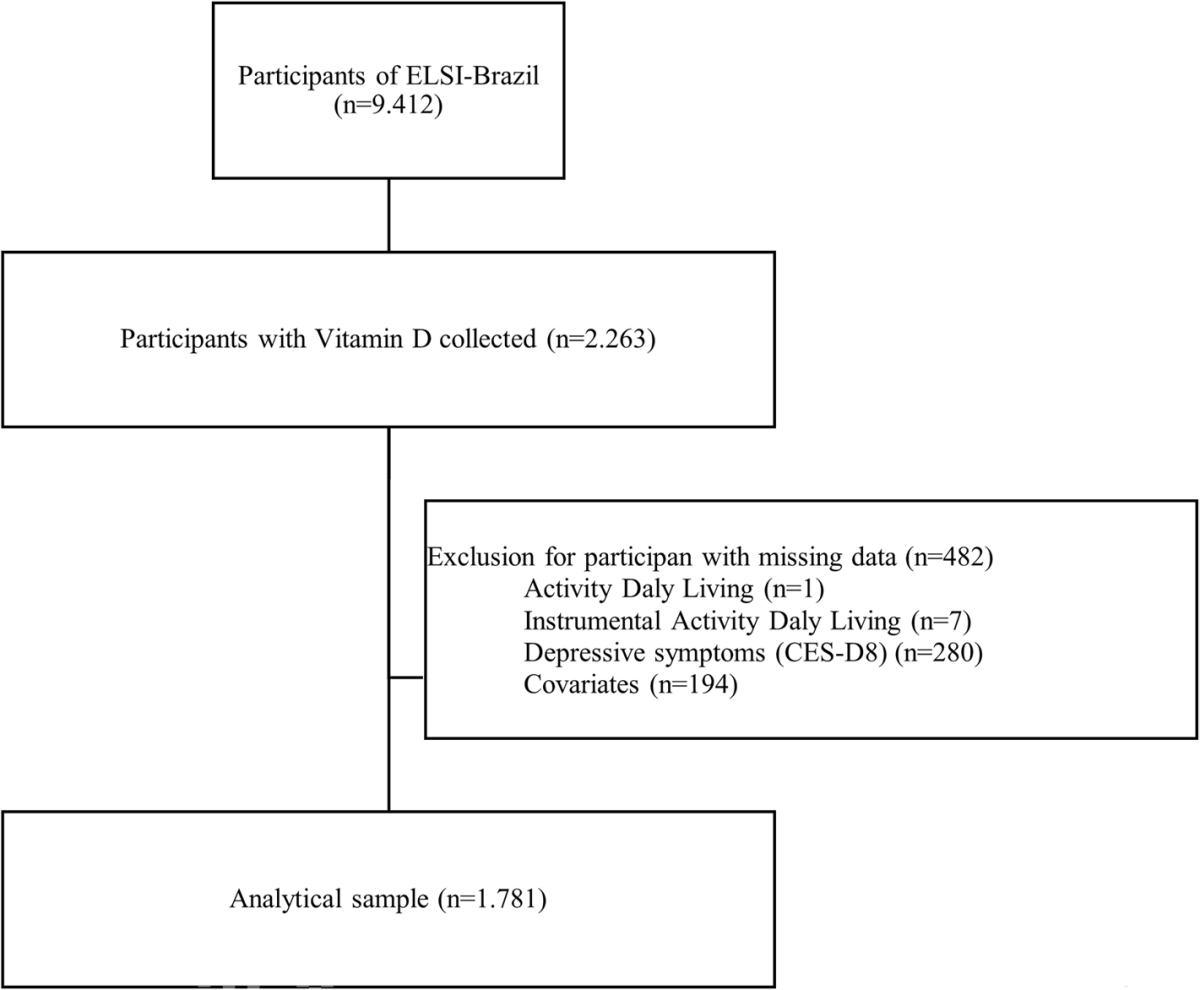
The flow chart of the participants’ selection, ELSI-Brazil, 2015–16.

For the present study, ELSI-Brazil participants with complete information aged 50 and over, who underwent blood collection and with complete data, were included. Individuals aged 50 years were included because changes inherent to aging start from the fifth decade of life, especially in developing countries. The total number of Elsi-Brazil participants is 9412. Participants aged 80 years and over were excluded due to the low sample size (n = 104). The other reasons for exclusion were presented in Fig. 1 (n = 7527). The analysis sample of the present study was 1781 participants.

### Outcome variable

Functional disability was independently investigated through two variables: difficulty in performing ADL (bathing, dressing, eating, being continent, having sexual activities, and sleeping independently) and in performing IADL (taking the bus, shopping, managing finances, managing medication use, and using the phone)^31^. The responses to the variables were classified as either having no difficulty or difficulty in one or more activities for those who reported difficulty or need help in at least one activity^32,33^.

### Exposure variables

The exposure variables were the vitamin D insufficiency and the presence of depressive symptoms. Four groups based on a combination of vitamin D insufficiency depressive symptoms were created: (1) sufficient vitamin D and no depressive symptoms; (2) sufficient vitamin D and depressive symptoms; (3) vitamin D insufficiency and no depressive symptoms, and; (4) vitamin D insufficiency and depressive symptoms.

Vitamin D was collected by a trained laboratory technician at the participant’s home while fasting. All biological samples were sent to the central laboratory at Oswaldo Cruz Foundation^30^ located in São Paulo and, depending on the distance, they were sent by air. The samples were stored on dry ice and during transport, the temperature was constantly monitored. A chemiluminescent microparticle immunoassay with an automatic analyser was used to measure the serum level of 25(OH)D, whose analytical sensitivity is 8.5 nmol/L, and the coefficient of variation ranged from 5.8 to 6.2%^30,31^. Regarding vitamin D levels, it was considered insufficient (< 30 nmol/L) and sufficient (≥ 30 nmol/L)^34,35^.

Depressive symptoms were assessed using the short version of the CES-D-8 scale^36^. The eight questions referred to the week before the interview and participants answered “yes” or “no” to the presence of a certain symptom. The score ranged between 0 and 8 and, in the present study, ≥ 4 symptoms were classified as the presence of depressive symptoms and ≤ 3 symptoms as the absence of depressive symptoms^37^.

### Adjustment variables

The following adjustment variables were used for this analysis: age (in years), sex (female and male), skin color (white, brown, black, yellow, indigenous), education in years (no formal education, 1 to 4 years, 5 to 8 years, 9 to 11 years, ≥ 12 years), marital status (with or without a partner), per capita income (in tertiles), nutritional status by body mass index (BMI) [low weight (< 18.5 kg/m^2^), normal (≥ 18.5 to 25 kg/m^2^), overweight (≥ 25 to 30 kg/m^2^), obesity (≥ 30 kg/m^2^)], physical activity level [active (≥ 150 min) and insufficiently active (< 150 min)]^38–40^, cognitive function^31^ (z score of ≤ − 1SD considered with cognitive impairment^41^), geographic region of residence (North, Northeast, Midwest, Southeast, and South) and season of the year during vitamin D collection (summer, autumn, winter, and spring).

### Statistical analysis

All analyses were performed using the *Stata SE 16 software*, considering the sample weights due to the sample selection. The descriptive analysis was presented in relative frequency with 95% confidence intervals (95% CI). For continuous data, measures of central tendency and dispersion were used. A bivariate analysis was performed between the dependent variables, difficulty in ADL and IADL, and the independent variables, and the test χ^2^ was adopted to test associations, in categorical variables, and Kruskal–Wallis test for continuous variables.

Crude and adjusted Poisson regression, with respective 95% CI, were used to investigate measures of association between outcome and exposures. After the adjusted analysis, the interaction of the combination of vitamin D insufficiency and depressive symptoms with age was tested using the post-estimation with the *margins* command, which is presented graphically.

### Ethical approval

The ELSI-Brazil was approved by the Research Ethics Committee of the Oswaldo Cruz Foundation (FIOCRUZ), Minas Gerais, with Certificate of Presentation for Ethical Consideration (CAAE) number 34.649.814.0000.5091. All participants interviewed in the study consented by signing a free and informed consent form to participate in the study.

## Results

Of the 9412 ELSI participants at baseline, only 2263 had vitamin D samples collected. 482 participants were excluded due to missing data (1 in ADL, 7 in IADL, 280 in CES-D-8, and 194 in covariates), totalling 1,781 participants included in the present analysis (Fig. 1). The mean age of the participants was 63.4 years (SD = 9.2). Most were women (52.1%), had a partner (56.9%), with 1 to 4 years of education (37.4%), were from the white race/ethnicity group (44.8%), upper income tertile (37.8%), physically active (68.7%) and, overweight (36.0%).13.0% had below 1SD of the memory score, most participants were from the Southwest and vitamin D was collected in spring. Overall, 59.7% had insufficient vitamin D levels, 33.2% had increased depressive symptoms, 14.6% had difficulties with ADL, and 47.9% in IADL (Table 1).

**Table 1:** Descriptive analysis and prevalence of functional disability in basic activities of daily living (ADL) and instrumental activities of daily living (IADL), ELSI-Brazil, 2015–2016.

Variable	n	% (95% CI)	ADL		IADL	
			% (95% CI)	*p*†-value	% (95% CI)	*p*†-value
**Vitamin D Level**				0.344		0.263
Sufficient	749	40.3 (33.8;47.1)	13.1 (9.0;18.6)		45.1 (37.5;52.8)	
Insufficient	1032	59.7 (52.9;66.2)	15.6 (11.9;20.2)		49.8 (41.5;58.1)	
**Depressive symptoms**				< 0.001		< 0.001
Absence (≤ 3 symptoms)	1158	66.8 (62.9;70.5)	9.9 (6.9;14)		39 (32.3;46.1)	
Presence (≥ 4 symptoms)	623	33.2 (29.5;37.2)	24 (18.8;30)		65.8 (57.4;73.3)	
**Vitamin D and depressive symptoms combination**				< 0.001		< 0.001
Sufficient vitamin D and no depressive symptoms	486	27.6 (24.0;31.4)	10.5 (6.2;17.1)		35.2 (27.8;43.4)	
Vitamin D insufficiency and no depressive symptoms	263	12.7 (9.0;17.8)	18.8 (12.1;28.0)		66.4 (57.4;74.3)	
Sufficient vitamin D and depressive symptoms	672	39.2 (33.6;45.1)	9.5 (6.4;14.0)		41.6 (33.2;50.6)	
Vitamin D insufficiency and depressive symptoms	360	20.5 (17.7;23.7)	27.2 (21.1;34.2)		65.4 (54.4;75.1)	
**Age (years)**	1781	63.4 (9.2)‡	65.5 (10.7) ‡	0.002*	65.0 (9.8)	< 0.001*
**Sex**				< 0.001		< 0.001
Female	1140	52.1 (47.8;56.3)	15.2 (11.5;19.8)		55.0 (48.0;61.9)	
Male	641	47.9 (43.7;52.2)	14.0 (10.2;18.7)		40.2 (32.1;48.9)	
**Marital status**				0.768		0.656
With partner	1066	56.9 (61.5;40.0)	14.8 (11.1;19.5)		48.4 (40.8;56.1)	
Without partner	715	34.1 (30.0;38.5)	14.1 (10.4;18.9)		46.9 (39.3;54.6)	
**Skin color**				0.570		0.778
White	708	44.8 (36.7;53.1)	12.5 (8.1;18.7)		45.2 (36.7;54.1)	
Black	179	9.5 (6.6;13.6)	20.0 (9.8;36.4)		48.6 (35.7;61.7)	
Brown	825	43.1 (37.4;48.9)	15.3 (11.9;19.4)		50.3 (41.9;58.7)	
Yellow	22	1.2 (0.6;2.4)	14.6 (3.2;47.0)		49.9 (19.0;80.9)	
Indigenous	47	1.5 (0.7;3.1)	23.0 (5.3;61.5)		51.9 (25.4;77.3)	
**Education**				< 0.001		< 0.001
12 years or more	128	7.1 (5.0;9.9)	7.9 (3.8;15.8)		35.6 (22.9;50.7)	
9 to 11 years	355	26.2 (21.4;31.7)	6.4 (3.1;12.9)		27.9 (18.3;40.1)	
5 to 8 years	351	19.1 (16.2;22.3)	14.3 (9.5;20.9)		47.6 (37.1;58.4)	
1 to 4 years	730	37.4 (32.0;43.1)	18.6 (14.0;24.3)		59.3 (51.8;66.4)	
No formal education	217	10.3 (7.8;13.5)	25.7 (15.4;39.8)		66.3 (54.2;76.5)	
**Income**				0.109		< 0.001
Upper tertile	596	37.8 (31.7;44.4)	11.2 (7.4;79.0)		36.1 (28.4;44.7)	
Second tertile	570	29.3 (25.2;33.8)	17.0 (12.4;22.9)		60.4 (52.3;67.8)	
Lower tertile	615	32.9 (26.2;40.3)	16.3 (11.8;22.0)		50.4 (39.2;61.5)	
**Physical activity level**				< 0.001		0.149
Active	1193	68.7 (63.4;73.6)	11.7 (8.6;15.9)		45.4 (38.6;52.5)	
Insufficient	588	31.3 (26.4;36.6)	20.8 (15.4;27.6)		53.3 (41.9;64.4)	
**Body mass index**				0.009		0.246
Normal	471	27.5 (23.8;31.6)	11.7 (7.8;17.0)		43.4 (32.8;54.6)	
Low weight	40	2.2 (1.5;3.1)	39.1 (19.7;62.1)		59.4 (36.1;79.1)	
Overweight	693	36.0 (32.0;40.1)	14.6 (11.1;19.1)		46.8 (40.2;53.5)	
Obesity	577	34.3 (31.4;37.4)	15.3 (10.9;21.2)		51.9 (43.2;60.5)	
**Cognitive function**				0.940		0.405
Normal (> − 1SD)	1540	87.0 (83.7;89.7)	14.6 (11.3;18.6)		47.2 (41.1;43.4)	
Cognitive impairment (≤ − 1SD)	241	13.0 (10.3;16.3)	14.8 (8.7;24.1)		52.8 (36.5;68.5)	
**Geographic region**				0.482		0.582
North	168	3.4 (1.2;9.3)	10.5 (6.1;17.6)		52.2 (43.3;60.9)	
Northeast	678	26.0 (14.6;42.1)	19.2 (13.5;26.6)		48.6 (37.4;60.0)	
Southwest	504	42.9 (27.2;60.3)	14.5 (10.0;20.6)		50.7 (41.9;59.5)	
South	256	20.0 (7.9;42.4)	10.9 (3.3;30.4)		38.8 (18.5;63.9)	
Midwest	175	7.6 (2.4;21.4)	10.6 (5.1;20.8)		51.5 (40.0;62.8)	
**Season**				0.122		0.227
Summer	158	5.9 (3.6;9.8)	9.2 (4.6;17.8)		48.8 (37.9;59.8)	
Autumn	121	8.5 (5.1;14.0)	10.6 (5.5;19.4)		39.6 (28.0;52.5)	
Winter	614	20.8 (11.6;34.5)	20.2 (13.5;29.2)		56.2 (47.4;64.7)	
Spring	888	64.7 (51.6;75.9)	13.8 (9.9;18.8)		46.2 (36.3;56.5)	
**Basic activity of daily living**						
No difficulty	1512	85.4 (81.4;88.7)				
One or more difficulties	269	14.6 (11.3;18.6)				
**Instrumental activity of daily living**						
No difficulty	858	52.1 (45.2;59.0)				
One or more difficulties	923	47.9 (41.0;54.8)				

^†^*p*-value of the chi-square test (*χ*^2^). * Kruskal–Wallis *p*-value. ‡Mean (SD).

In the bivariate analysis, there was a significantly higher prevalence of those who had difficulties in performing ADL among women, those with no formal education, those who were physically inactive, those with low BMI, with the presence of depressive symptoms, and those with insufficient vitamin D. Regarding IADL difficulties, the prevalence was significantly higher in women, in those with low education, and in those without depressive symptoms and with vitamin D insufficiency (Table 1).

In the crude analysis, the presence of depressive symptoms alone and combined with vitamin D insufficiency were associated with ADL disability. This association remained significant in the fully adjusted model. The combination of depressive symptoms with vitamin D insufficiency increases by 2.20 (95% CI: 1.25; 3.86) the prevalence of ADL disability to those who had sufficient levels of vitamin D and no depressive symptoms (Table 2).

**Table 2:** Crude and adjusted analysis of functional disability in basic activities of daily living (ADL) and instrumental activities of daily living (IADL), according to depressive symptoms, vitamin D insufficiency, and a combination of both, ELSI-Brazil, 2015–2016.

Variable	Crude	Adjusted*
	PR (95% CI)	PR (95% CI)
**ADL**		
**Depressive symptoms**		
Absence (≤ 3 symptoms)	1	1
Presence (≥ 4 symptoms)	**2.41 (1.71; 3.41)**	**2.19 (1.52; 3.15)**
**Vitamin D**		
Sufficient	1	1
Insufficient	1.18 (0.83; 1.70)	1.13 (0.81; 1.57)
** Vitamin D and depressive symptoms combination**		
Sufficient Vitamin D and no depressive symptoms	1	1
Vitamin D insufficiency and no depressive symptoms	1.79 (0.96; 3.32)	1.61 (0.91; 2.82)
Sufficient Vitamin D and depressive symptoms	0.90 (0.52; 1.58)	0.84 (0.49; 1.46)
Vitamin D insufficiency and depressive symptoms	**2.59 (1.53; 4.39)**	**2.20 (1.25; 3.86)**
**IADL**		
**Depressive symptoms**		
Absence (≤ 3 symptoms)	1	1
Presence (≥ 4 symptoms)	**1.69 (1.46; 1.95)**	**1.49 (1.29; 1.72)**
**Vitamin D**		
Sufficient	1	1
Insufficient	1.11 (0.93; 1.32)	1.04 (0.88; 1.22)
**Vitamin D and depressive symptoms combination**		
Sufficient Vitamin D and no depressive symptoms	1	1
Vitamin D insufficiency and no depressive symptoms	**1.89 (1.49; 2.39)**	**1.62 (1.35; 1.95)**
Sufficient Vitamin D and depressive symptoms	1.18 (0.92; 1.52)	1.09 (0.87; 1.37)
Vitamin D insufficiency and depressive symptoms	**1.86 (1.53; 2.26)**	**1.54 (1.24; 1.91)**

*Analysis adjusted for sex, age, marital status, race, education, per capita income, physical activity, body mass index, memory score, region of residence, and season of the year during vitamin D collection.

Significant values are in bold.

Regarding IADL disability, the presence of depressive symptoms was associated with the presence of difficulties in carrying out activities in the crude and fully adjusted analysis, with a 49.0% increase in prevalence (PR: 1.49; 95% CI: 1.29; 1.72). Vitamin D insufficiency was not associated with IADL. When depressive symptoms were combined with vitamin D insufficiency, both in the crude and adjusted analyses, being insufficient in vitamin D levels alone was associated with a 62% increase in disability in the adjusted analysis (PR: 1.62; 95% CI: 1.35; 1.95). The combination of depressive symptoms and vitamin D insufficiency increased the prevalence by 54% (PR: 1.54; 95% CI: 1.24; 1.91), while sufficient levels of vitamin D and depressive symptoms were not associated with IADL disability (Table 2).

**Figure 2:**
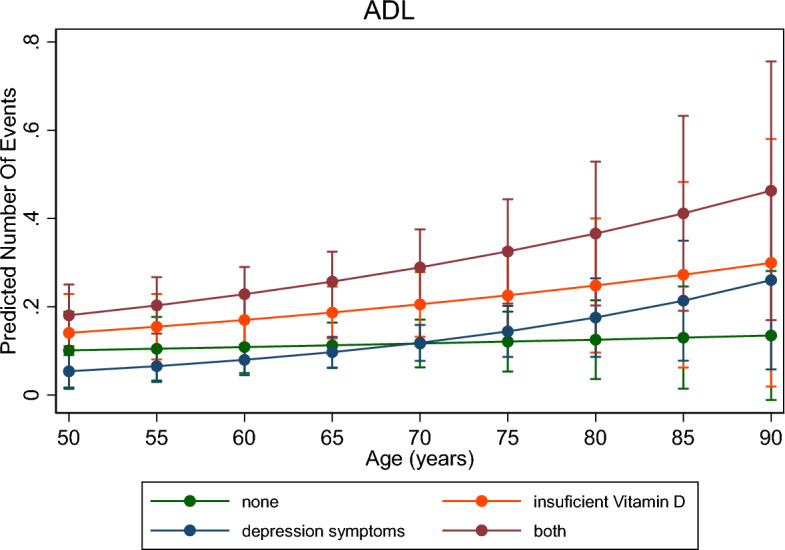
Interaction between ADL and age according to the combination of vitamin D levels and depressive symptoms, ELSI-Brazil, 2015–2016.

Figure 2 shows the interaction of the vitamin D insufficiency and depressive symptoms with age about ADL. There is an increase in the prevalence of functional disability with increasing age, but it occurs differently for those who have only depressive symptoms and sufficient levels of vitamin D.

**Figure 3:**
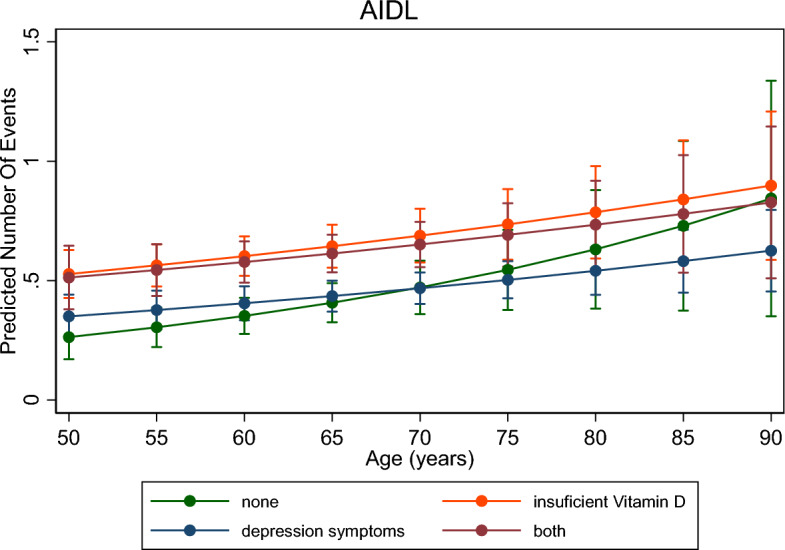
Interaction between IADL and age according to the combination of vitamin D levels and depressive symptoms, ELSI-Brazil, 2015–2016.

Figure 3 shows the evolution of IADL with aging. Overall, there is an increase in functional disability concerning age for all vitamin D and depressive symptoms groups. However, for those who have both conditions and insufficient vitamin D alone, the IADL increase is greater than in the other groups.

## Discussion

The key findings of the present study showed that vitamin D insufficiency and, mainly, the concomitant presence of depressive symptoms increase the prevalence of functional disability, thus compromising the performance of both ADL and IADL. The prevalence of functional disabilities has increased with age. However, this increase did not occur to the same extent for ADL and IADL concerning the combination of vitamin D status and the presence of depressive symptoms.

The study by Koning et al.^29^, with old Dutch adults, showed that 17.0% of the participants had vitamin D deficiency. Depressive symptoms affected 15.1% of the sample at the beginning of the study and after six years it increased to 19.8%. In a cross-sectional analysis, there was no significant association between vitamin D and depressive symptoms. However, in a longitudinal analysis, older women with vitamin D concentrations less than 75 nmol/L had 17.0% to 24.0% more depressive symptoms than women with vitamin D concentrations greater than 75 nmol/L. The Brazilian older adults in the present study showed higher levels of vitamin D insufficiency and the presence of elevated depressive symptoms. However, the association with reduced functionality was similar.

Rafiq et al.^42^ showed that participants with lower levels of vitamin D were older, with worse physical performance and more depressive symptoms. Chronic diseases, depressive symptoms, and physical performance proved to be mediators in explaining the relationship between vitamin D and quality of life. The characterization of the study sample and the categorization values were also different from our study, as they considered levels < 25 nmol/L as deficiency, from 25 to 50 nmol/L as insufficiency, and > 50 nmol/L as sufficient, not allowing a proper comparison. However, functional worsening was present in those with advanced age, with vitamin D insufficiency and depressive symptoms, as in the present study.

The presence of low vitamin D levels has been reported^26^ as a cause for the functional decrease due to the appearance of muscle weakness, osteomalacia, hyperparathyroidism, and, consequently, increased bone reduction, favouring the onset of osteoporosis and osteopenia. These factors cause less mobility and a higher incidence of falls and fractures. On the other hand, functional disability makes it impossible for vitamin D to perform its functions actively and effectively^43^. In the present study, our sample showed no impairment in ADL, as they were active and functional, but when depressed, having the association of vitamin D insufficiency with depressive symptoms further increased functional impairment.

Depressive symptoms can increase the chance of ADL and IADL impairment^44^, as depressive symptoms can lead to an inactive lifestyle^45,46^ and to reinforce the damage caused by vitamin D insufficiency, through reduced by sun exposure and lower nutritional consumption combined with the decrease in nutrient metabolism, commonly reduced during aging^47^.

The opposite relationship is also addressed by authors, in which vitamin D deficiency is considered a cause of presence of depressive symptoms^26,27^. The presence of vitamin D receptors in places in the brain where serotonin is produced explains the onset of cognitive impairment, depressive symptoms, and anxiety after vitamin D insufficiency^27,48^.

More than half of the participants in our study did not report elevated depressive symptoms. A possible explanation for this low percentage is that our sample had a younger population and, therefore, the tendency to present the factors that influence the onset of depressive symptoms was smaller. Because they do not present the functional impairments that affect the oldest old, our sample also presented the characteristic of being active and functional. Being physically active is a protective factor for depression and, similarly, the more functional, with a higher level of physical activity, the lower the risk of depressive symptoms^49^.

It is usual to find Brazilians and internationals studies with older adult and the results point to vitamin D insufficiency. Even in the sunniest regions, low serum levels of this vitamin are common, as just the presence of sunlight alone is not enough. In addition, the skin of the older adults loses effectiveness, further reducing the ability to synthesize vitamin D through sun exposure. Another factor that can decrease the serum levels of vitamin D in later life is being overweight, as the increase in body fat makes it difficult to distribute vitamin D due to the decrease in bioavailability^47^.

We investigated the magnitude of the increase in disability at all ages and its relationship with depressive symptoms and vitamin D insufficiency. The greater disability observed could be explained, since presence of depressive symptoms often increases with age^50^, as physiological changes affect physical functioning, compromising basic skills such as sitting, walking, getting up; and, consequently, the quality of life, due to low self-esteem and increased depressive symptoms^51^. As for vitamin D, the aging process can cause a decline in renal function, consequently decreasing the renal production of 1,25(OH)2D^47^. Also, with advancing age, the concentration of the intestinal vitamin D receptor decreases, signalling that it is one of the causes of resistance to 1,25(OH)2D in the, consequently leading to a decrease^52^.

Previous studies carried out in various states of Brazil found characteristics that are like our study as a profile of the older adults: being female^53,54^, having a partner^53,55^, and having low education^56^. Data from the Brazilian Institute of Geography and Statistics (IBGE) also confirm that women and the white race are the majority among the elderly^57^. What justifies this finding is the characteristic that follows the aging process of having a greater number of long-lived women than men. In this regard, the United Nations (UN) estimate is that by 2040 there will be 6.2 million more women than men^58^.

In our research, we included the age group between 50 and 59 years to follow the beginning of the aging process. However, in some studies^54–56^, the participants included are those over 60 years of age, and the age group between 60 and 69 years prevailed. Although this study presents very important points, its limitations should be highlighted. Firstly, the type of study does not allow establishing a temporal relationship. Secondly, there is the bias of non-participants that causes sample loss in the study because the weakest tend not to participate. Third, the instruments used are widely used in other studies and allow comparisons with research at a global level, but they are self-reported measures and can be influenced by social, environmental and psycho-emotional factors, in addition to being subject to memory or information bias. Although vitamin D insufficiency alone has caused little impairment in functionality, it is necessary to maintain sufficient serum levels of vitamin D, because, regardless of the initial factor, whether depressive symptoms or even other possible physical condition impairments that arise from the aging process, the loss of function will become even greater if an older adult is impaired. Therefore, strategies to keep this population active and with good levels of vitamin D are important. Including those at risk of hypovitaminosis D and contraindications to sun exposure, vitamin D supplementation needs to be considered.

Daily activities, such as walking to the market, and keeping the house clean and organized, among others, are very important for maintaining the elderly’s independence. However, access to physical activity is essential for the aging process to take place with quality. Cooperating with the maintenance of serum levels of vitamin D, physical activities can be performed outdoors and in groups, encouraging social interaction. Integrative, multitasking, playful, and recreational practices can also be added to contribute not only to the prevention/recovery of depression but also to cognitive maintenance.

The maintenance and recovery of functional capacity resulting from the interaction of multiple factors, physical and mental health, conditions to independently perform common daily tasks, participation in community life, economic independence, being able to count on family support, and having access to health care professionals. Likewise, depressive symptoms are not just a physiological response that emerges during the aging process, but a multifactorial consequence that needs to be carefully observed.

We recommend that new studies investigate the longitudinal relationship between depressive symptoms and vitamin D on functionality, considering the region of residence and the season of data collection. Consider an analysis that considers categories such as deficiency, insufficiency and sufficiency of vitamin D. It is worth noting that Brazil is a country of large dimensions, climate and means of subsistence. In this sense, the availability of food itself differs in each region, which is directly related to vitamin D. In the present study, we did not include data on nutrition, but we reinforce the suggestion for future studies.

### Final considerations

The concomitant presence of vitamin D insufficiency and depressive symptoms increases the chances of functional disability in adults. Healthcare professionals can prioritize the early identification and treatment of vitamin D deficiencies and depressive symptoms in adult patients to help reduce the risk of functional disability.

## Data Availability

The data can be accessed at https://elsi.cpqrr.fiocruz.br/data-access/
